# Adjunctive Aripiprazole Versus Placebo for Antipsychotic-Induced Hyperprolactinemia: Meta-Analysis of Randomized Controlled Trials

**DOI:** 10.1371/journal.pone.0070179

**Published:** 2013-08-01

**Authors:** Xianbin Li, Yilang Tang, Chuanyue Wang

**Affiliations:** 1 Beijing Key Lab of Mental Disorders, Beijing Anding Hospital, Capital Medical University, Beijing, China; 2 Department of Psychiatry and Behavioral Sciences, Emory University School of Medicine, Atlanta, Georgia, United States of America; Baylor College of Medicine, United States of America

## Abstract

**Objective:**

To compare the safety and efficacy of adjunctive aripiprazole versus placebo for antipsychotic-induced hyperprolactinemia.

**Methods:**

Population: adult patients presenting with antipsychotic-induced hyperprolactinemia diagnosed by prolactin level with or without prolactin-related symptoms. Interventions: adjunctive aripiprazole vs. adjunctive placebo. Outcome measures: adverse events and efficacy of treatment. Studies: randomized controlled trials.

**Results:**

Five randomized controlled trials with a total of 639 patients (326 adjunctive aripiprazole, 313 adjunctive placebo) met the inclusion criteria. Adjunctive aripiprazole was associated with a 79.11% (125/158) prolactin level normalization rate. Meta-analysis of insomnia, headache, sedation, psychiatric disorder, extrapyramidal symptom, dry mouth, and fatigue showed no significant differences in the adjunctive aripiprazole treatment group compared with the placebo group (risk difference (Mantel-Haenszel, random or fixed) −0.05 to 0.04 (95% confidence interval −0.13 to 0.16); I^2^ = 0% to 68%, P = 0.20 to 0.70). However, sedation, insomnia, and headache were more frequent when the adjunctive aripiprazole dose was higher than 15 mg/day. Meta-analysis of the prolactin level normalization indicated adjunctive aripiprazole was superior to placebo (risk difference (Mantel-Haenszel, random) 0.76 (95% confidence interval 0.67 to 0.85); I^2^ = 43%, P<0.00001). The subgroup analysis confirmed that the subjects who received adjunctive aripiprazole 5 mg/day showed a degree of prolactin normalization similar to that of all participants. No significant differences between groups in discontinuation and improvements of psychiatric symptoms.

**Conclusion:**

Adjunctive aripiprazole is both safe and effective as a reasonable choice treatment for patients with antipsychotic-induced hyperprolactinemia. The appropriate dose of adjunctive aripiprazole may be 5 mg/day.

## Introduction

Hyperprolactinemia is a common and serious side effect of antipsychotic treatment [1]. Hyperprolactinemia is an apparent challenge in the treatment of antipsychotic-induced adverse effects, particularly in female patients, with a prevalence between 42%–66% [2]. Hyperprolactinemia has both short and long-term consequences including menstrual irregularities, amenorrhea, galactorrhea, gynecomastia, sexual dysfunction, infertility, osteoporosis, and even breast cancer [2]–[4].

Four strategies have been recommended for the treatment of this side effect. (1) Reduction of antipsychotic dose, but maintenance treatments with a reduction to a lower dose have a higher relapse rate than the no-dose-reduction treatment [5]. (2) Switching to a different antipsychotic agent, however this is not always possible if the patient has responded well to the current antipsychotic. Furthermore, the alternative antipsychotics may be associated with other adverse effects, such as sedation, weight gain, and diabetes [6]–[8]. (3) Adjunctive dopamine agonists, such as bromocriptine. Evidence suggests the addition of dopamine agonists often aggravates psychosis and abnormal involuntary movements [9]–[11]. (4) Adjunctive herbal medicine, although psychiatrists in China and Japan always add herbal medicine to manage antipsychotic-induced hyperprolactinemia in clinical practice [9], [12], only a few, small sample studies have been conducted to test the efficacy [9], [13]. While it is an interesting and potentially important adjunctive treatment, the findings so far are inconclusive and further studies are clearly needed in this field.

On the other hand, the role of aripiprazole in antipsychotic-induced hyperprolactinemia has recently gained some attention. Several case reports and a small open label trial showed the addition of aripiprazole reversed antipsychotic-induced hyperprolactinemia to risperidone [14], [15] as well as to paliperidone, sulpiride, amisulpride, and ziprasidone [16]–[18]. Randomized controlled trials indicated normalization in prolactin levels by adjunctive aripiprazole in patients maintained with risperidone, haloperidol and sulpiride treatment [19]–[21]. Although adjunctive aripiprazole is effective for hyperprolactinemia, combination therapy is not a standard practice [22] and it is not recommended by the practice guidelines for the treatment of schizophrenics [22]. Also, studies involving combination therapy have so far yielded conflicting findings regarding efficacy [23]–[26] and the majority of studies have shown that polypharmacy increased the incidence of adverse events [27]–[29].

As a newer pharmacologic atypical antipsychotic, adjunctive aripiprazole may optimize D2 receptor activity, and thus reduce risk for extrapyramidal symptoms associated with haloperidol or risperidone [30] and decrease prolactin elevation resulting from full antagonists [28]. Thus, adjunctive aripiprazole may have additional benefits in both efficacy and side effects [20]. Though there have been quite a few randomized controlled trials of adjunctive aripiprazole for hyperprolactinemia, the findings are inconsistent and sometimes conflicting. Some studies indicated that the addition of aripiprazole was generally safe and well tolerated [20], [31], [32], while others found that adjunctive aripiprazole was associated with increased adverse events, including sedation, insomnia, headache, etc. [20], [21]. So far, there has been no exhaustive system reviews on this topic.

The aim of this meta-analysis of randomized controlled trials was to compare adjunctive aripiprazole treatment with placebo for the treatment of antipsychotic-induced hyperprolactinemia, with particular focus on safety and tolerability.

## Methods

### Types of Studies

Randomized controlled trials comparing adjunctive aripiprazole with placebo for antipsychotic-induced hyperprolactinemia in adult patients were eligible for inclusion. We included studies which reported at least one of the outcome measures mentioned below and with well defined treatment programs. We excluded case series, retrospective studies, non-randomized studies and system reviews.

### Types of Outcome Measures

We recorded clinical outcomes based on intent to treat (ITT) analysis if available. The primary outcome measures of this system review were adverse events and efficacy of treatment, as reported in the studies (Table 1). The secondary outcome measures were discontinuation and improvement in psychiatric symptoms (Table 1). The results of statistical heterogeneity were presented in the forest plots.

**Table 1:** Definition of outcome measures.

Outcome measure	Adjunctive aripiprazole or placebo
Adverse events	**Included adverse events:** Extrapyramidal symptoms, Sedation, Dry mouth, Fatigue, Insomnia, Headache, Psychiatric disorders
Prolactin level normalization	Hyperprolactinemia defined as prolactin level ≥60 µg/L, normal prolactin level was defined as a serum prolactin level of <30 ng/ml for the patients in Chinese study. Normal prolactin level was defined as a serum prolactin level of <24 ng/ml for women and <20 ng/ml for men in the study by shim.
Prolactin-related symptoms recovery	**Included prolactin-related symptoms:** Oligomenorrhea, Amenorrhea, Galactorrhea
Discontinuation rate	All reasons for study discontinuation were included in meta- analysis.
Improvement in psychiatric symptoms	**Measurements:** In the change of the Positive and Negative Syndrome Scale total score of adjunctive aripiprazole compared with placebo in two studies; and the Brief Psychiatric Rating Scale score in three studies.

### Selection of Studies

Two authors independently searched the Pubmed, Embase, and Cochrane Library databases for randomized controlled trials comparing adjunctive aripiprazole with placebo for antipsychotic-induced hyperprolactinemia. We also searched the Chinese databases (CBM and CNKI databases) using the same key words. The search included all studies published between January 2001 and December 2012, regardless of language. Keywords used included: aripiprazole, hyperprolactinemia, prolactin abnormal, randomized controlled trial, controlled clinical trial, randomized, placebo, drug therapy, randomly, and trial. The keywords were used in combination with the Boolean operators AND, OR, and NOT. We used the “related article” function to supplement the search. We manually searched bibliographies of randomized controlled trials for studies that were missed in the electronic search. Two authors assessed studies that met the inclusion criteria for the meta-analysis independently; the third author would be involved in discussion if any disagreements existed.

### Assessment of Risk of Bias in Included Studies

We used the method of random sequence generation (selection bias), allocation concealment (selection bias), blinding of participants and personnel (performance bias), blinding of outcome assessment (detection bias), incomplete outcome data (attrition bias), selective reporting (reporting bias), and other bias to assess the methodological quality of included individual randomized controlled trials. Furthermore, we used the grading of recommendations assessment, development, and evaluation (GRADE) system to rate the quality of evidence and strength of recommendations of this meta-analysis. This was recommended by the Cochrane Collaboration. GRADE included systematic assessments of all included trials across six main domains for each outcome: limitations of the study design and execution, inconsistency, indirectness, and imprecision of results, publication bias, and large treatment effect. Accordingly, we graded the recommendation for outcome measure of either adjunctive aripiprazole or placebo as very low, low, moderate, or high.

### Date Extraction

We performed the meta-analysis according to the recommendations of the Cochrane Collaboration, using the Review Manager Version 5.1.7.0 software. Two authors independently extracted data for analysis. We presented the summary statistic of dichotomous outcomes as risk difference for adverse events, discontinuation, and treatment efficacy. The Mantel-Haenszel and Inverse-Variance methods were used to combine the summary statistic. We used I^2^ method to assess the statistical heterogeneity. When combining studies for meta-analysis, we assumed that variation existed between trials, so we used a random effect model to provide a conservative estimate of the results if I^2^>25% [33]. We used a fixed effect model if no heterogeneity existed. All statistical differences were considered significant when the p<0.05.

### Subgroup Analysis

We conducted separate subgroup analysis for adverse events, discontinuation and prolactin level normalization of Chinese studies in this meta-analysis. The dose of aripiprazole was all 5 mg/day in Chinese studies, and the definition of hyperprolactinemia was prolactin level >60 µg/L in all of these studies. We did this subgroup analysis to examine if variations in populations, antipsychotics dosage and definition of hyperprolactinemia across studies may have been responsible for the heterogeneity of the primary outcome. We assessed whether these differences influenced the outcome.

## Results

### Quality Assessment

**Figure 1:**
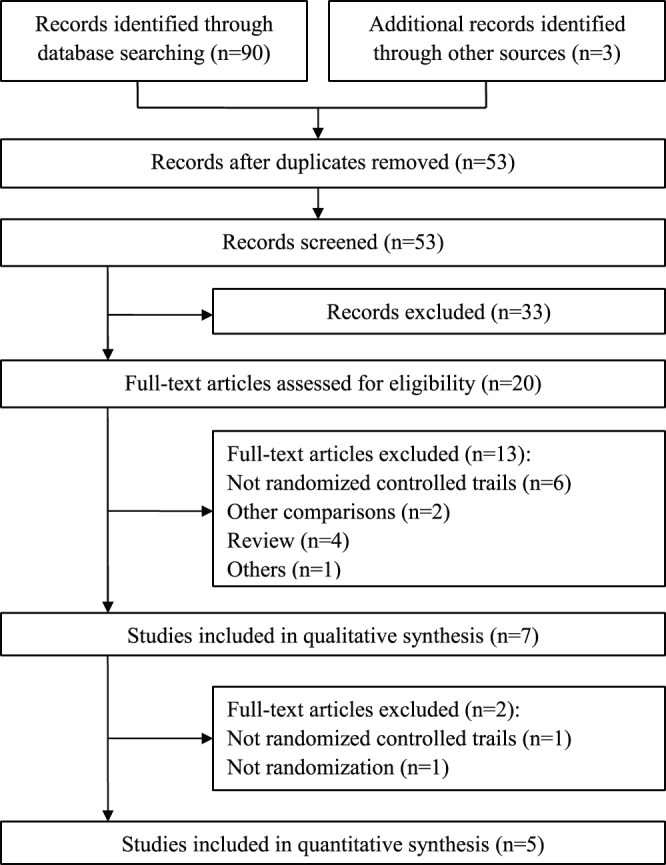
PRISMA flow diagram.

We initially considered seven trials and included five in our analysis (Figure 1). Table 2 summarized the characteristics of the five included studies. Of the two excluded, one study was not blind, and the other one did not report method of randomization. Four studies included patients treated in one center; another one was a multi-center study. The diagnosis of hyperprolactinemia was made from the laboratory tests for serum prolactin level (Table 1), in addition there were two studies reported the prolactin-related adverse events.

**Table 2:** Individual study.

Xu 2006
***Methods:*** Randomized, single-blind, placebo-controlled study.
***Participants:*** All 60 female patients with schizophrenia, aged 18–35 years with chronic, stable condition. Who had serum prolactin levels ≥60 µg/L and oligomenorrhea or galactorrhea 3 months after risperidone or sulpiride treatment were enrolled. Patients were ineligible if they had any vital medical condition, especially endocrine disease, and had pregnancy within two years.
***Interventions:*** Participants received adjunctive aripiprazole 5 mg daily or placebo for 6 weeks respectively.
***Outcomes:*** Decreases in mean serum prolactin levels, the ratio of prolactin level normalization, and the incidence of side effects.
Shim 2007
***Methods:*** Randomized, double-blind, placebo-controlled study
***Participants:*** Fifty-four patients with schizophrenia, either gender, ages 18 to 45, clinically stable, who had been treated with haloperidol monotherapy and were taking the same dosage of haloperidol for at least 3 months. Other eligibility requirements included the presence of hyperprolactinemia, no past history of drug or alcohol abuse, and no medical and/or neurological illness.
***Interventions:*** Participants were randomly assigned to receive aripiprazole or placebo treatment. Aripiprazole was dosed at 15 mg/day for the first 4 weeks, and then increased to 30 mg/day for the following 4 weeks if it was clinically tolerated.
***Outcomes:*** Decreases in mean serum prolactin levels, the ratio of prolactin level normalization, and the incidence of side effects.
Ji 2008
***Methods:*** Randomized, single-blind, placebo-controlled study.
***Participants:*** All 130 female patients with schizophrenia, aged 18–35 years, with chronic, stable condition. Who had serum prolactin levels ≥60 µg/L after fixed dose risperidone treatment were enrolled. Patients were ineligible if they had any endocrine disease, and had pregnancy within two years.
***Interventions:*** Participants received adjunctive aripiprazole 5 mg daily or placebo for 6 weeks respectively.
***Outcomes:*** Decreases in mean serum prolactin levels, the decline rate and the normalization ratio of prolactin level, the incidence of side effects.
Chen 2009
***Methods:*** Double-blind, randomized, placebo-controlled study.
***Participants:*** Seventy-two male patients with schizophrenia, ages 18 to 50, never taking antipsychotic drugs. All the patients were treated with risperidone for 4 weeks, and then patients whose prolactin level reached to 60 ug/L were enrolled. Patients were ineligible if they had any vital medical condition, drug or alcohol abuse and significant abnormal laboratory test.
***Interventions:*** Participants were administered additional aripiprazole 5 mg daily or placebo for 8 weeks
***Outcomes:*** Decreases in mean serum prolactin levels, the decline rate and the ratio of prolactin level normalization, the incidence of side effects.
Kane 2009
***Methods:*** Randomized, double-blind, placebo-controlled study in 43 American sites.
***Participants:*** Outpatients of either gender, age≥18 years with chronic, stable schizophrenia or schizoaffective disorder and currently receiving a stable dose of quetiapine (400–800 mg/d) or risperidone (4–8 mg/d) for≥4 weeks but with an inadequate response were entered. Patients were ineligible if they had any medically significant abnormal laboratory test or vital sign.
***Interventions:*** Participants were randomly assigned to receive aripiprazole (2–15 mg/d) or placebo for 16-week adjunctive therapy.
***Outcomes:*** Improvement in psychiatric symptoms, decreases in serum prolactin levels, the ratio of prolactin level normalization, the incidence of side effects.

**Figure 2:**
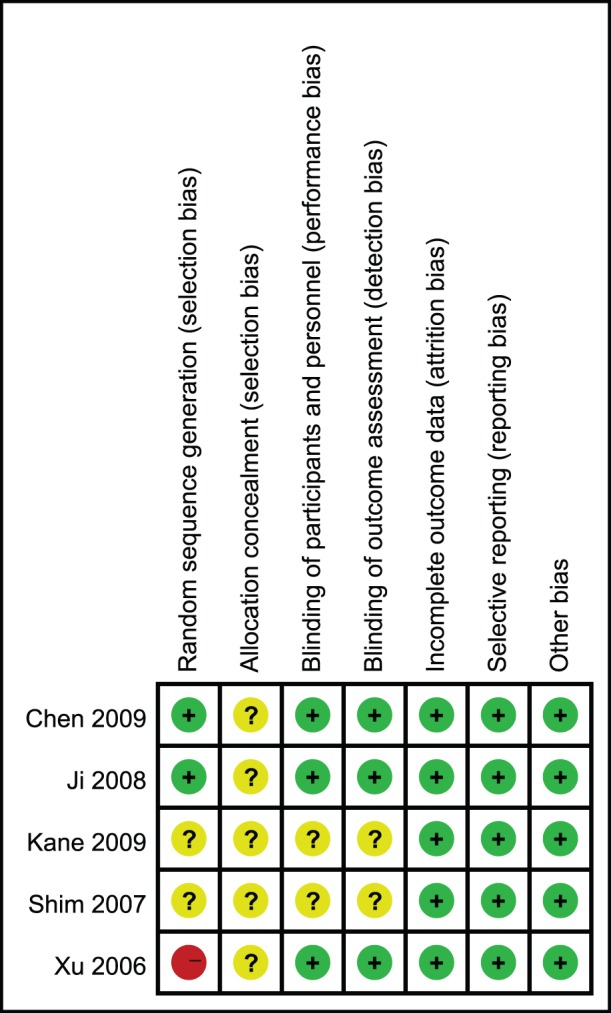
Risk of bias summary: review authors' judgments about each risk of bias item for each included study.

Randomization methods were reported as computer generated in one study, and random number table generated in another studies. The randomization method was high risk in another study because baseline adaptive random grouping was reported, which was partial randomization. Concealment of allocation was all unclear risk in five studies because it was not reported. Blinding methods were unclear in two studies, the rest of the studies were low risk in blinding. All studies described dropouts and withdrawals (Figure 2).

### Analysis of Outcomes

Table 3 and Table 4 summarized outcomes for individual randomized controlled trials. A large number of patients were on adjunctive aripiprazole 5 mg/day. Therefore, we did a secondary analysis excluding data from the study with aripiprazole dose >5 mg/day to avoid the risk of any selection bias and over-estimation of treatment effects. Quality assessment of the included studies according to the GRADE approach showed some limitations of the study design and inconsistency, and some strengths of large treatment effect, no obvious indirectness or imprecision of reporting of results. Based on the above assessments, the quality of evidence presented for each outcome ranged from “low” to “high” (Table 5). We did not conduct a funnel plot analysis to show the presence of risk of publication bias, because only five studies included, asymmetry could not be reliably judged. Nonetheless, we did not identify any unpublished negative studies related to the papers.

**Table 3:** Summary of studies.

Study	Trail Design	Participants	Weeks	N	Antipsychotic	Aripiprazole dose(mg/day)	Rate of prolactin level normalization	Prolactin-related symptom recovery
Xu 2006	Placebo-controlled,single-blind	Female	6	60	Risperidone Sulpiride	5	Aripiprazole: 83.3%Placebo: 0%	27/28 patients regained menstruation; 16/16 no longer complained galactorrhea; No change in placebo group.
Shim 2007	Placebo-controlled,double-blind	Either gender	8	54	Haloperidol	15–30	Aripiprazole: 88.5%Placebo: 3.6%	7/11 patients regained menstruation; 1/2 no longer complained galactorrhea; No change in placebo group.
Ji 2008	Placebo-controlled,single-blind	Female	6	130	Risperidone	5	Aripiprazole: 82.0%Placebo: 4.0%	_
Chen 2009	Placebo-controlled,double-blind	Male	8	72	Risperidone	5	Aripiprazole: 67.6%Placebo: 6.5%	_
Kane 2009	Placebo-controlled,double-blind	Either gender	16	322	Risperidone Quetiapine	5–15	Aripiprazole: 19.5%Placebo: 9.1%	_

**Table 4:** Summary of adverse events and discontinuation.

Study	Averse events(incidence rate: % or the number of events: n)	Discontinuation(incidence rate: % or the number of events: n)
Xu 2006	**Aripiprazole:** Headache: 3%, Insomnia: 3%, Sedation: 10%;**Placebo:** Insomnia: 13%	_
Shim 2007	**Aripiprazole:** Insomnia: 42%, Dry mouth: 31%, Headache:23%, Sedation: 12%, Weakness: 8%, Psychiatric disorder: 7.7%;**Placebo:** Dry mouth: 21%, Sedation: 18%, Insomnia: 18%	Two patients in the aripriprazole group experienced insomnia, anxiety, and irritability, and both opted for withdrawal from the study.
Ji 2008	**Aripiprazole:** Headache: 3, Insomnia: 3, Sedation: 6;**Placebo:** Headache: 4, Insomnia: 5	Adjunctive aripiprazole vs. placebo: Subject was lost to follow-up (2 vs. 4); Switching to other antipsychotics(3 vs. 4);
Chen 2009	**Aripiprazole:** Dry mouth: 3, Sedation: 2, Tremor: 4, Anxiety: 4,Constipation: 6, Salivate: 8, tachycardia: 8, ALT goes up: 5, Elevatedglucose level: 3, ECG ST segment changes: 3; **Placebo:** Dry mouth: 3,Sedation: 3, Tremor: 4, Anxiety: 5, Tachycardia: 6, Constipation: 4,Salivate: 6, ALT goes up: 6, ECG ST segment changes: 4,Elevated glucose level: 3.	Adjunctive aripiprazole vs. placebo: Adverse event (1 vs. 1); Subject was lost to follow-up (0 vs. 1); Switching to other antipsychotics(2 vs. 1);
Kane 2009	**Aripiprazole:** Fatigue: 14(8.3%), Headache: 12(7.1%), Insomnia:11(6.5%), Akathisia: 10(5.9%), Somnolence: 10(5.9%), Psychiatricdisorder: 2(1.2%), Back pain: 10(5.9%); **Placebo:** Fatigue:10(6.5%), Headache: 13(8.5%), Insomnia: 13(8.5%), Akathisia: 11(7.2%),Somnolence: 7(4.6%), Psychiatric disorder: 15(9.8%),Back pain: 4(2.6%)	Adjunctive aripiprazole vs. placebo: Adverse event (5.4% vs. 10.3%); Subject withdrew consent (8.9% vs. 5.8%); Subject was lost to follow-up (7.1% vs. 7.7%); Poor/noncompliance (7.1% vs. 3.2%); Other reasons (2.4% vs. 3.9%); Lack of efficacy (0.6% vs. 0%).

**Table 5:** GRADE Analysis: quality assessment of adjunctive aripiprazole versus placebo for antipsychotic-induced hyperprolactinemia.

Critical outcome	Participants(studies)	Risk ofbias*	Inconsistency *	Largeeffect^$^	imprecision	Public bias	Overall quality of evidence^#^
**Insomnia:** All studies	566(4)	Serious^ a, b^	Serious ^c^	No	No	undetected	+/+/−/−/; low
Studies with aripiprazole 5 mg/day	190(2)	Serious^ a, b^	No	No	No	undetected	+/+/+/−/; moderate
**Headache:** All studies	566(4)	Serious ^a, b^	Serious ^c^	No	No	undetected	+/+/−/−/; low
Studies with aripiprazole 5 mg/day	190(2)	Serious^ a, b^	No	No	No	undetected	+/+/+/−/; moderate
**Sedation:** All studies	638(5)	Serious ^a, b^	No	No	No	undetected	+/+/+/−/; moderate
Studies with aripiprazole 5 mg/day	262(3)	Serious^ a, b^	No	No	No	undetected	+/+/+/−/; moderate
**Psychiatric disorder:**	448(3)	No	Serious ^c^	No	No	undetected	+/+/+/−/; moderate
**Extrapyramidal symptom:**	394(2)	No	No	No	No	undetected	+/+/+/+/; high
**Dry mouth:**	126(2)	No	No	No	No	undetected	+/+/+/+/; high
**Fatigue:**	376(2)	No	No	No	No	undetected	+/+/+/+/; high
**Prolactin level normalization:** All studies	316(4)	Serious ^a, b^	No	Very large^ d^	No	undetected	+/+/+/+/; high
Studies with aripiprazole 5 mg/day	262(3)	Serious ^a, b^	Serious ^c^	Very large^ d^	No	undetected	+/+/+/+/; high
**Discontinuation:** All studies	638(5)	Serious ^a, b^	No	No	No	undetected	+/+/+/−/; moderate
Studies with aripiprazole 5 mg/day	262(3)	Serious ^a, b^	No	No	No	undetected	+/+/+/−/; moderate

* **Decrease quality of evidence:** a) single-blind method; b) randomization by the antipsychotic weight; c). I^2^>50%; **^$^Increase quality of evidence:** d) RR >5 or <0.2; **GRADE Working Group grades of evidence: # High quality:** Further research is very unlikely to change our confidence in the estimate of effect. **Moderate quality:** Further research is likely to have an important impact on our confidence in the estimate of effect and may change the estimate. **Low quality:** Further research is very likely to have an important impact on our confidence in the estimate of effect and is likely to change the estimate. **Very low quality:** We are very uncertain about the estimate.

### Adverse Events

**Figure 3:**
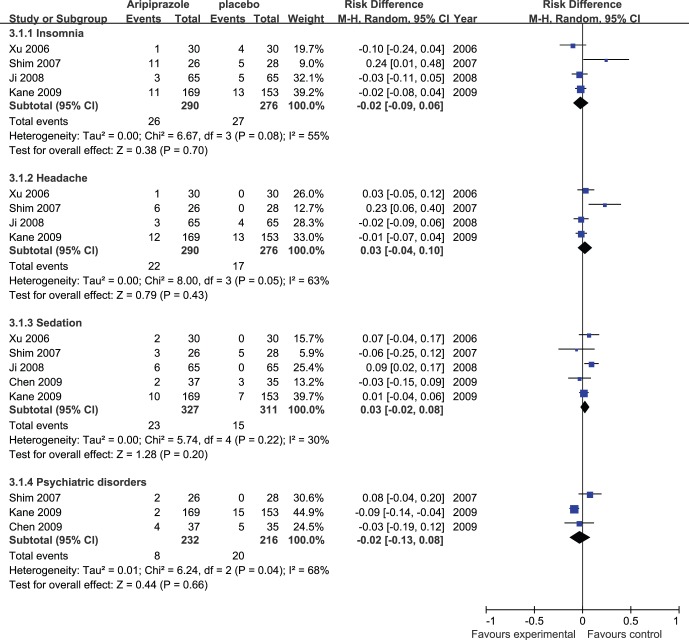
Adjunctive aripiprazole vs. placebo for antipsychotic-induced hyperprolactinemia: forest plot for insomnia, headache, sedation, and psychiatric disorder.

Meta-analysis of insomnia, headache, sedation, and psychiatric disorder (Figure 3) showed no significant differences in the adjunctive aripiprazole treatment group compared with the placebo group (risk difference (Mantel-Haenszel, random) −0.02 to 0.03 (95% confidence interval −0.13 to 0.10); I^2^ = 30% to 68%, P = 0.20 to 0.70).

**Figure 4:**
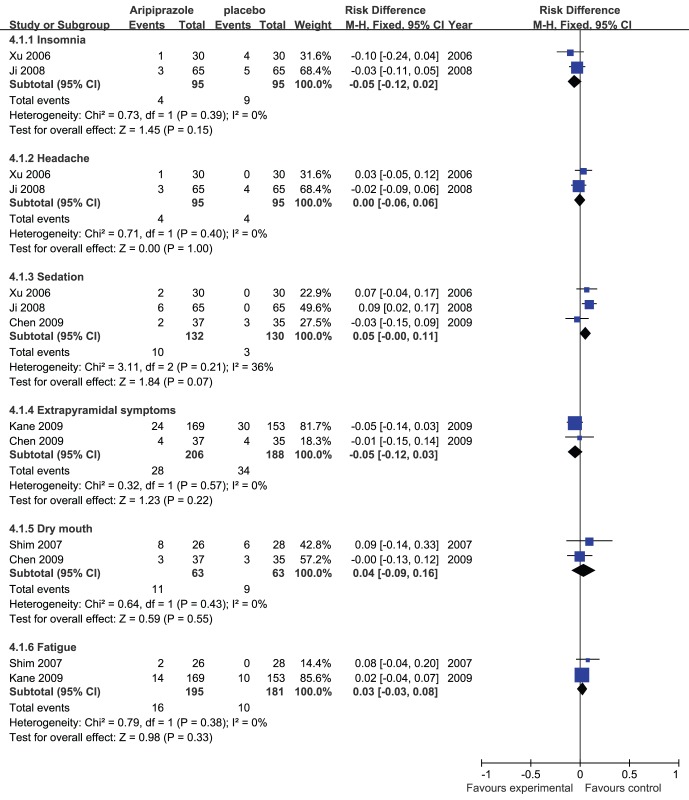
Adjunctive aripiprazole vs. placebo for antipsychotic-induced hyperprolactinemia: forest plot for a secondary analysis of insomnia, headache, sedation and forest plot for extrapyramidal symptoms, dry mouth, and fatigue.

A secondary analysis of insomnia, headache, and sedation including the data from the studies with adjunctive aripiprazole dose 5 mg/day (Figure 4) also showed no significant differences in the adjunctive aripiprazole treatment group compared with the placebo group (risk difference (Mantel-Haenszel, fixed) −0.05 to 0.05 (95% confidence interval −0.12 to 0.11); I^2^ = 0% to 36%, P = 0.07 to 1.00).

Meta-analysis of extrapyramidal symptoms, dry mouth, and fatigue (Figure 4) showed no significant differences between the two groups (risk difference (Mantel-Haenszel, fixed) −0.05 to 0.04 (95% confidence interval −0.12 to 0.16); I2 = 0%, P = 0.22 to 0.55).

### Treatment Efficacy

**Figure 5:**
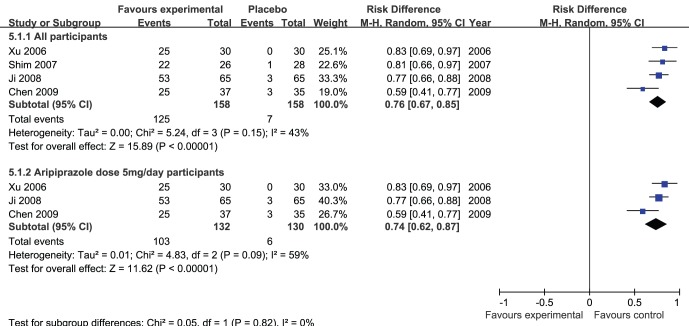
Adjunctive aripiprazole vs. placebo for antipsychotic-induced hyperprolactinemia: forest plot for prolactin level normalization.

Meta-analysis of the prolactin level normalization (Figure 5) showed adjunctive aripiprazole was superior to placebo (risk difference (Mantel-Haenszel, random) 0.76 (95% confidence interval 0.67 to 0.85); I^2^ = 43%, P<0.00001).

A secondary analysis of the prolactin level normalization including the data from the studies with adjunctive aripiprazole dose 5 mg/day (Figure 5) also indicated that adjunctive aripiprazole was superior to placebo(risk difference (Mantel-Haenszel, random) 0.74 (95% confidence interval 0.62 to 0.87); I^2^ = 59%, P<0.00001).

Two studies reported the change of prolactin-related symptoms (Table 2). In one study, 7 of 11 (63.60%) female patients having menstrual disturbances regained menstruation, and 1 of 2 patients (50%) no longer complained symptoms of galactorrhea in adjunctive aripiprazole group (Table S2). In another study, 27 of 28 (96.43%) patients regained menstruation, and 16 of 16 (100%) patients no longer complained symptom of galactorrhea in aripiprazole group. No patients in the placebo group showed fully recovery in prolactin-related symptoms in the two studies.

### Discontinuation

**Figure 6:**
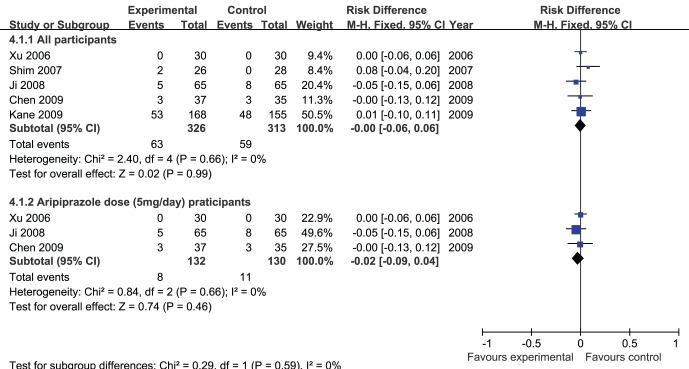
Adjunctive aripiprazole vs. placebo for antipsychotic-induced hyperprolactinemia: forest plot for discontinuation.

Meta-analysis of discontinuation (Figure 6) showed no significant differences in the adjunctive aripiprazole treatment group compared with the placebo group (risk difference (Mantel-Haenszel, fixed) −0.00 (95% confidence interval −0.06 to 0.06); I^2^ = 0%, P = 0.99).

A secondary analysis of discontinuation including the data from the studies with adjunctive aripiprazole dose 5 mg/day (Figure 6) also showed no significant differences in the adjunctive aripiprazole treatment group compared with the placebo group(risk difference (Mantel-Haenszel, fixed) −0.02 (95% confidence interval −0.09 to 0.04); I^2^ = 0%, P = 0.46).

### Improvement in Psychiatric Symptoms

In all of the five studies, there were differences in the mean change from baseline to endpoint in either PANSS or BPRS total score in the adjunctive aripiprazole treatment group compared with the placebo group. (Table 1, Figure S1). Meta-analysis of improvement in psychiatric symptoms (Figure S1) showed no significant differences in three studies (mean difference (Inverse-Variance, fixed) −0.46 (95% confidence interval −1.13 to 0.20); I^2^ = 0%, P = 0.17) between the two groups.

## Discussion

This first meta-analysis of five randomized controlled trials including 639 patients compared the safety and efficacy of adjunctive aripiprazole treatment with that of adjunctive placebo in antipsychotic-induced hyperprolactinemia. It showed that adjunctive aripiprazole is generally safe and well tolerated, with no significant increase in the risk of adverse events and discontinuation compared with placebo. No significant differences in insomnia, headache, sedation, psychiatric disorder, extrapyramidal symptoms, dry mouth, and fatigue were found. Furthermore, adjunctive aripiprazole was superior to placebo in prolactin level normalization. However, augmentation with aripiprazole failed to demonstrate significant improvement in psychiatric symptoms compared with placebo.

### Strengths of the Study

To the best of our knowledge, this is the first study to review the safety of adjunctive aripiprazole treatment. Adverse events occurred at similar incidence rates among patients receiving adjunctive aripiprazole and placebo, except for sedation, insomnia, and headache, which were more frequent in the adjunctive aripiprazole (dose >15 mg/day)group than in the placebo groups. These side effects are known to occur in a small proportion of patients given aripiprazole [20]. Thus we recommend that when one considers adjunctive aripiprazole treatment, attention should be paid to monitoring for symptoms of sedation, insomnia, and headache. Furthermore, the incidence of discontinuation was comparable between adjunctive aripiprazole groups and placebo groups. The results of these analysis showed that adjunctive aripiprazole 5–10 mg/day is safe and well tolerated for the treatment of antipsychotic-induced hyperprolactinemia. Adjunctive aripiprazole’s favorable safety and tolerability profile may lead to improved compliance in the management of hyperprolactinemia. The low rates of adverse events in adjunctive aripiprazole may attribute the pharmacodynamic profile of aripiprazole: potent partial agonist activity at dopamine D2 receptors stabilizes the dopamine system while avoiding the hypodopaminergia that may limit the tolerability of currently available antipsychotics [34]. Although aripiprazole is a partial agonist at D2, clinical deterioration or worsening of symptoms generally did not occur with adjunctive aripiprazole treatment for hyperprolactinemia in this meta-analysis.

Although most case reports, open label studies, and placebo-controlled trials in adult patients have shown that adjunctive aripiprazole is associated with lower prolactin levels, unchanged or even elevated prolactin levels have also been reported by some studies [34]. Our meta-analysis indicated that adjunctive aripiprazole significantly lowered prolactin level in patients with antipsychotic-induced hyperprolactinemia. Consistent with the improvements in prolactin levels, after adjunctive aripiprazole treatment, 37 participants with menstrual disturbances regained menstruation, 17 patients no longer complained of signs or symptoms of galactorrhea in two studies, whereas no patients did so in the placebo group. The prolactin-lowering effect of aripiprazole is likely due to its unique pharmacology as a D2 receptor partial agonist. With haloperidol or risperidone treatment, antagonist activity at D2 receptors in the tuberoinfundibular system region reduces dopamine activity, increasing the risk of hyperprolactinemia. In contrast, aripiprazole may act as a dopamine agonist in conditions of low endogenous dopamine activity [35], [36], which prevents the development of hypodopaminergia in the tuberoinfundibular system region, thereby decreasing the serum prolactin level.

We also searched Chinese database in this meta-analysis, which included randomized controlled trials comparing adjunctive aripiprazole with placebo for antipsychotic-induced hyperprolactinemia in China. Thus, it is the first study to include all trials available without applying any language restrictions. Moreover, we conducted subgroup analysis of Chinese studies in this meta-analysis. This subgroup analysis confirmed the significant superiority for adjunctive aripiprazole 5 mg/day versus placebo regarding the prolactin level normalization; furthermore we also confirmed that there was no significantly difference in insomnia, headache, sedation, and discontinuation between adjunctive aripiprazole 5 mg/day and placebo. Thus, the finding suggest that 5 mg/day dose of adjunctive aripiprazole may be appropriate, in fact adverse events were more frequent when adjunctive aripiprazole dose >15 mg/day in previous discussion.

### Limitations of the Study

There was significant heterogeneity of the results in adverse events and prolactin level normalization in this meta-analysis, suggesting the effect of relevant moderator and mediator variables. This may be because meta- analyses combine results from trials that differ in their methodology, study size and year, patient and treatment selection, outcome variables, and study conduct. However, when combining studies of this nature for meta-analysis, we used a random effect model to provide a conservative estimate of prolactin level normalization and adverse events. We used a fixed effect model to analyze discontinuation because no heterogeneity existed. On the other hand, we conducted subgroup analysis of Chinese studies in this meta-analysis that sought to disentangle relevant moderator variables, strengthening the primary finding. In addition, in the meta-analysis of the prolactin level normalization, the study by Kane et al. (2009) was excluded because the presence of hyperprolactinemia was not an eligibility criterion.

This meta-analysis only included 639 patients in five randomized controlled trials. The sample size was relatively small for meta-analysis, which prevented further data exploration. However, we included studies with well defined randomized controlled trials comparing adjunctive aripiprazole with placebo. Although there were about 10 randomized controlled trials published in Chinese language, only 3 trials were included in this meta-analysis after a more strict quality assessment. In addition, random sequence generation was high risk in one study, potentially contributing to selection bias; however, this study was low risk in other bias. Although the GRADE approach showed the quality of evidence were “low” in insomnia and headaches, other outcomes were from “moderate” to “high”, especially the quality of evidence in prolactin level normalization were all “high”. This high quality of evidence will increase our confidence to use adjunctive aripiprazole to manage antipsychotic-induced hyperprlactinemia.

The correlation between aripiprazole dose and the change in prolactin levels may not have been fully analyzed because the aripiprazole doses were all higher than 5 mg/day in this meta-analysis. In contrast, in a previous study, 2 mg/day of aripiprazole acted as a partial dopamine agonist producing a clinical effect [20], [37], [38]. It suggests the possibility that doses lower than 5 mg/day of aripiprazole can significantly decrease prolactin levels. The association between aripiprazole dose and prolactin level when used as adjunctive treatment needs to be more fully evaluated.

### Comparison with other Studies

A review by Hoffer et al. (2009) included case studies, reports, and placebo-controlled trials studies to study the effectiveness of aripiprazole in lower prolactin level [39]. Across all studies, aripiprazole reduced prolactin levels on an average of 74.30%, which is comparable to our meta-analysis. However, on the basis of this analysis, the adverse events were not reported in the adjunctive aripiprazole treatment. Secondly, this review just reviewed each study on a case-by-case basis and reported mean prolactin levels, although they computed mean percent-reductions in prolactin level in 16 studies, they did not conduct any quality assessment. Furthermore, this review failed to include Chinese studies, as well as the study by Shim et al (2009).

Marder et al. (2003) conducted a pooled analysis of safety and tolerability data from five 4- to 6-week double-blind multi-center studies of patients with schizophrenia or schizoaffective randomized to aripiprazole (n = 932) or placebo (n = 416) [34]. This analysis indicated that aripiprazole was well tolerated, with similar AE incidence rates to placebo. The median percentage changes from baseline at endpoint in serum prolactin levels were significantly lower for the aripiprazole group compared with the placebo group (56.5% vs. 0.0%), which is consistent with our meta-analysis. Unfortunately, all adjunctive aripiprazole studies were not included in this analysis.

Similarly, Deleon and Citrome had reviewed the efficacy and tolerability of aripiprazole in schizophrenia and bipolar disorder respectively [40], [41]. Aripiprazole exhibited a favorable safety and tolerability profile, with a low propensity to cause extrapyramidal side effects, weight gain and hyperprolactinemia. Aripiprazole and other antipsychotics are commonly used for the initial treatment of acute mania, both as monotherapy and as adjuncts to mood stabilizers [42], [43]. Aripiprazole may be the appropriate adjunctive antipsychotic for the patients with bipolar who have high risk for hyperprolactinemia.

### Implications for Practice

Antipsychotic-induced hyperprolactinemia has both short and long-term consequences including menstrual irregularities, amenorrhea, galactorrhea, gynecomastia, sexual dysfunction, infertility, osteoporosis, and even breast cancer [44], [45]. Adjunctive aripiprazole treatment as the management of antipsychotic-induced hyperprolactinemia is safe and effective. Rapid adjunctive therapy will increase the compliance of patients with antipsychotic-induced hyperprolactinemia, which may offer benefits in schizophrenia treatment, and thus is a reasonable choice for antipsychotic-induced hyperprolactinemia.

### Conclusions

This meta-analysis of five randomized controlled trials comparing adjunctive aripiprazole and placebo have shown that adjunctive aripiprazole can be used safely and effectively for antipsychotic-induced hyperprolactinemia. Adjunctive aripiprazole is not associated with increased adverse events and discontinuations compared with placebo. Adjunctive aripiprazole is superior to placebo in prolactin level normalization. The recommended dose of adjunctive aripiprazole may be 5 mg/day. An early adjunctive aripiprazole to manage hyperprolactinemia may increase treatment compliance, which may offer benefits in schizophrenia treatment. The efficacy of adjunctive dose <5 mg/day needs further evaluation.

## Supporting Information

Figure S1 **Adjunctive aripiprazole vs. placebo for antipsychotic-induced hyperprolactinemia: forest plot for improvement in psychiatry symptom.**
(EPS)
